# Collapse of left lung after endotracheal intubation: Is it always due to misplacement of tube?

**DOI:** 10.4103/0974-2700.66532

**Published:** 2010

**Authors:** Mohan Gurjar, Sanjay Singhal, Banani Poddar, R K Singh

**Affiliations:** Department of Critical Care Medicine, Sanjay Gandhi Post Graduate Institute of Medical Sciences, Lucknow-226 014, India

**Figure 1:**
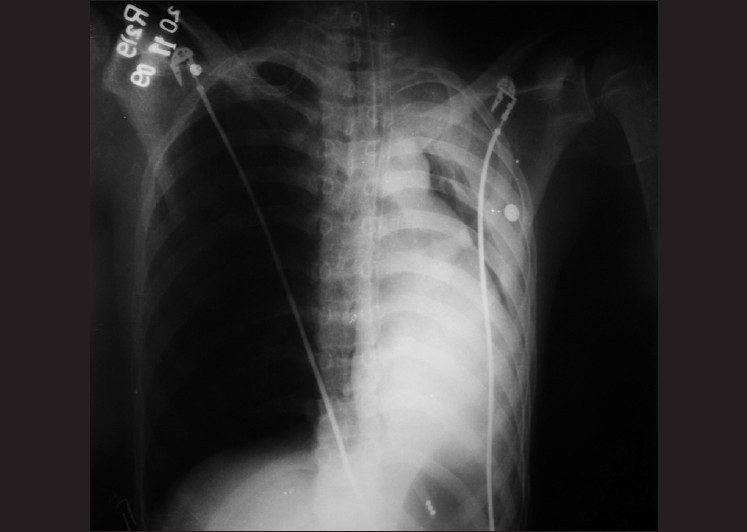
Left lung collapse due to mucus plug obstructing airways

Sir,

A 16-year-old boy was diagnosed to have Guillain-Barré syndrome, and admitted to ICU with respiratory failure secondary to respiratory muscle weakness. On examination, the respiratory rate was 30–35/min using accessory muscles along with decreased air entry in basal areas bilaterally. Neurologic evaluation revealed inability to swallow and poor coughing along with neuromuscular power of 1/5 in all the 4 limbs. Arterial blood gas analysis showed type 2 respiratory failure (on ventimask with FiO_2_ 0.4, pH 7.33, PaO_2_ 90.5, PaCO_2_ 47.8, HCO_3_‾ 24.6, Base Excess 1.7, SO_2_ 97.1%). The chest radiograph prior to admission was normal. Following admission to ICU, he was intubated (endotracheal tube size 8.0 mm and fixed at 21 cm) with adequate intravenous sedation (midazolam and fentanyl) and muscle relaxant (vecuronium); and the sedation maintained with continuous infusion of midazolam and fentanyl. The position of the endotracheal tube was confirmed by auscultation and chest expansion; and the patient was put on mechanical ventilation. A few minutes after intubation, his peripheral oxygen saturation decreased to 91%–93%. The position of the endotracheal tube was reconfirmed with direct laryngoscopy; however, the chest examination revealed decreased air entry on the left side of the chest. Arterial blood gas analysis showed hypoxemia (on FiO_2_ 0.4, pH 7.41, PaO_2_ 68.3, PaCO_2_ 41.4, HCO_3_‾ 25.7, Base Excess 1.0, SO_2_ 93.5%), while a postintubation chest radiograph revealed collapse of the left lung along with mediastinal shift toward the left and endotracheal tube at a proper position [Figure 1]. Unfortunately, a bronchoscope was not available at that time. In view of his normal chest radiograph prior to intubation, it was suspected that he might have mucus plug with left-sided lung collapse. By positioning the patient laterally and giving aggressive physiotherapy along with endotracheal suction, we were able to clear the mucus plug. Following this, he had marked improvement in his oxygenation and ventilation, which was reflected on the monitor.

Right main stem intubation is a common, because of the anatomy of right main bronchus, which may lead to acute left lung collapse. However, other reasons for acute lung collapse are kinking of tube, obstruction due to mucus plug, blood clot, or some foreign body. Significant lung collapse due to large mucus plugs occluding major airways have been described in many studies on lung diseases, such as cystic fibrosis, bronchiectasis, allergic bronchopulmonary aspergillosis, bronchial asthma, and so on.[1] Severe mucus plugging may lead to the worsening of gas exchange, increasing inspiratory pressure, and difficulty in breathing; and if not treated quickly, may lead to infectious complications, high morbidity, and mortality.[2]

Patients with neuromuscular disease involving respiratory muscles, despite having otherwise healthy lungs, suffer from insufficient airway clearance due to impaired mobility coupled with ineffective cough, which lead to pooled secretion and formation of mucus plug. This subgroup of patients behaves as having restrictive-lung disease resulting in shallow breathing, which limits the distribution of ventilation throughout the lobes of the lungs, and potentially leading to segmental atelectasis. These patients even when mechanically ventilated, are at a high risk because of impaired mucus transport and ineffective cough due to lack of adequate glottic closure.[3]

Maintaining good hydration and chest physiotherapy is considered a traditional and safe method for the treatment of atelectasis in the majority of these patients. Secretion management in the critically ill patients requiring mechanical ventilation includes passive or active humidification of inspired gases, percussion and postural drainage, and simulation of cough by manual hyperinflation or insufflation–exsufflation method, especially in those who have very weak cough.[4] Fiberoptic flexible bronchoscopy is indicated in patients who have excessive tracheobronchial secretion or suspected mucus plug with whole lung or nearly whole lung atelectasis to achieve lung expansion.[2]
